# XK-related protein 5 (XKR5) is a novel negative regulator of KIT/D816V-mediated transformation

**DOI:** 10.1038/s41389-018-0057-3

**Published:** 2018-06-18

**Authors:** Jianmin Sun, Tine Thingholm, Peter Højrup, Lars Rönnstrand

**Affiliations:** 1 Department of Pathogen Biology and Immunology, School of Basic Medical Sciences, Ningxia Medical University, Yinchuan, China; 2 Department of Laboratory Medicine, Translational Cancer Research, Lund University, Lund, Sweden; 3 Department of Laboratory Medicine, Lund Stem Cell Center, Lund University, Lund, Sweden; 4 Department of Biochemistry and Molecular Biology, University of Southern Denmark, Odense, Denmark; 5 Department of Oncology, Skåne University Hospital, Lund, Sweden

## Abstract

In order to investigate the molecular mechanisms by which the oncogenic mutant KIT/D816V causes transformation of cells, we investigated proteins that selectively bind KIT/D816V, but not wild-type KIT, as potential mediators of transformation. By mass spectrometry several proteins were identified, among them a previously uncharacterized protein denoted XKR5 (XK-related protein 5), which is related to the X Kell blood group proteins. We could demonstrate that interaction between XKR5 and KIT/D816V leads to phosphorylation of XKR5 at Tyr 369, Tyr487, and Tyr 543. Tyrosine phosphorylated XKR5 acts as a negative regulator of KIT signaling, which leads to downregulation of phosphorylation of ERK, AKT, and p38. This led to reduced proliferation and colony forming capacity in semi-solid medium. Taken together, our data demonstrate that XKR5 is a novel type of negative regulator of KIT-mediated transformation.

## Introduction

The stem cell factor receptor, KIT, is a receptor tyrosine kinase that has been found to be mutated and constitutively active in a number of human malignancies, including mast cell leukemia, core binding factor acute myeloid leukemia, gastrointestinal stromal tumors (GISTs), malignant melanoma and testicular carcinoma (for review, see ref.^1^). One of the most commonly found mutations, D816V, is located in the activation loop of the kinase domain. The exact mechanism by which it causes transformation is not fully understood. We and others have shown that KIT/D816V is not only constitutively active, but also has the capacity to phosphorylate other proteins than wild-type KIT^2–4^. In an attempt to gain further insight into the molecular pathways utilized by the KIT/D816V mutant, we immunoprecipitated either wild-type KIT or KIT/D816V from transfected Ba/F3 cells and analyzed the co-immunoprecipitating proteins. One of the proteins associating with KIT/D816V, but not with wild-type KIT, was a hitherto uncharacterized protein, XKR5 (XK-related protein 5). In this paper we demonstrate that XKR5 is a novel negative regulator of KIT signaling that inhibits KIT/D816V-induced transformation.

## Results

### XKR5 binds to the oncogenic mutant KIT/D816V but not to wild-type KIT

**Fig. 1:**
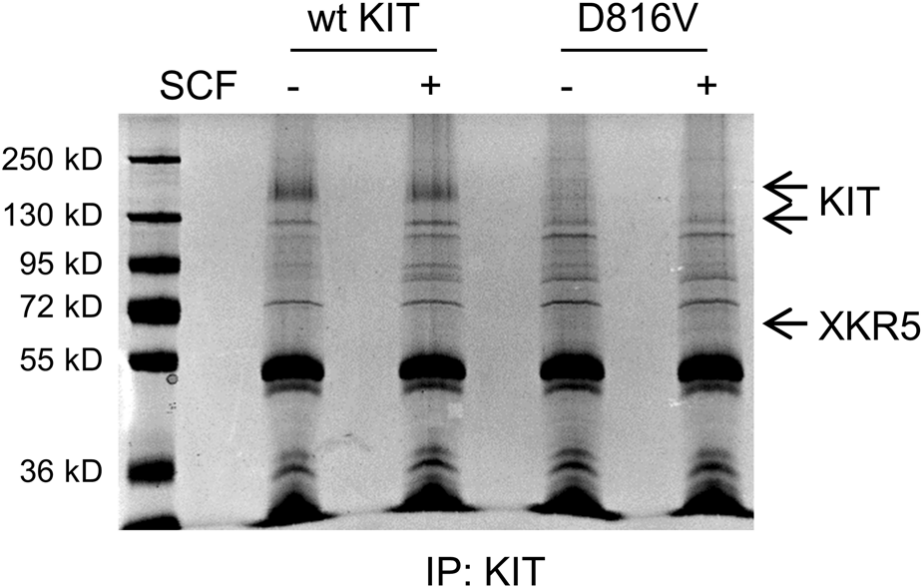
Identification of XKR5 as a protein selectively binding to KIT/D816V but not wild-type KIT. Nine hundred million Ba/F3 cells expressing either wild-type KIT or KIT/D816V were starved in medium without serum and IL-3 for 4 h followed by stimulation with SCF for 2 min. Cells were washed with PBS and lysed in lysis buffer. The lysates were centrifuged and supernatants were incubated with a KIT antibody for 1 h at 4 °C followed by incubation with protein G Dyna beads for 30 min at 4 °C. The immunoprecipitates were washed in lysis buffer, boiled for 5 min in SDS-PAGE sample buffer and separated by SDS-PAGE followed by staining with Coomassie Brilliant Blue. The band labeled “XKR5” was analyzed by mass spectrometry and found to be identical to XKR5

**Fig. 2:**
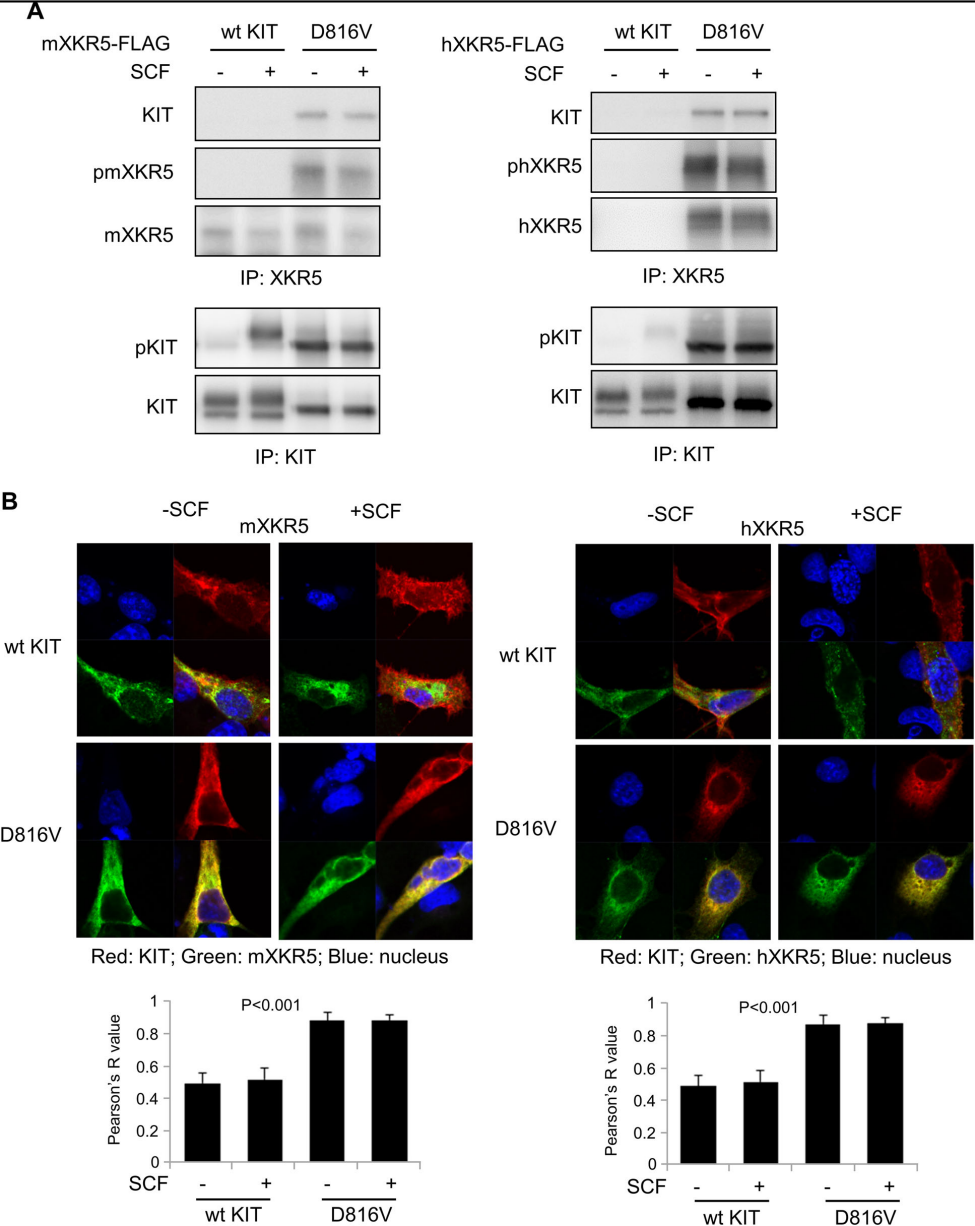
XKR5 binds to and colocalizes with KIT/D816V but not with wild-type KIT. **a** COS1 cells were co-transfected with FLAG-tagged XKR5 and wild-type KIT or KIT/D816V. 48 h after transfection, cells were stimulated with SCF for 2 min, lysed and immunoprecipitated with antibodies against FLAG-tag (for the detection of XKR5) or KIT antibody, respectively. After separation by SDS-PAGE and transfer to PVDF membranes, the membranes were probed with either KIT antibodies or phosphotyrosine antibodies to visualized phosphorylated proteins. **b** The localization of XKR5 and KIT was visualized by confocal microscopy. KIT was stained with a PE-conjugated antibody and XKR5-FLAG was stained with Alexa Fluor 647-conjugated FLAG antibody. The co-localization of XKR5 and KIT (three experiments) was quantified

It has been reported that the most commonly found activating KIT mutation, D816V, is not only constitutively active in the absence of ligand stimulation, but it also has gained an altered kinase specificity and therefore activates additional signaling pathways apart from those activated by wild-type KIT^5^. In order to study which additional signaling pathways that are activated by KIT/D816V, we purified KIT/D816V and its associated proteins by large scale immunoprecipitation from Ba/F3 cells expressing KIT/D816V. As a control, cells expressing wild-type KIT were used. We observed many additional bands in samples immunoprecipitated from KIT/D816V-expressing Ba/F3 cells compared to samples immunoprecipitated from wild-type KIT expressing cells (Fig. 1). This suggests that KIT/D816V utilizes additional proteins, apart from those used by wild-type KIT, to mediate its signals into the cell. The additional bands were excised and analyzed by mass spectroscopy. Several previously identified KIT binders were found (e.g., PI3-kinase) but also novel hitherto unknown KIT interactors. In order to verify our findings, we co-expressed several of these proteins in COS1 cells together with KIT/D816V and found that one of the proteins, that we could verify to associate with KIT/D816V, was the protein XKR5 (data not shown). As shown in Fig. 2a, both murine and human XKR5 were able to pull down KIT/D816V but not wild-type KIT, suggesting that XKR5 selectively associates with KIT/D816V but not with wild-type KIT. Colocalization of KIT/D816V with both murine and human XKR5 was demonstrated with confocal microscopy, while wild-type KIT did not show any co-localization with XKR5 (Fig. 2b). Thus, this further verifies that XKR5 is an interaction partner of KIT/D816V but not of wild-type KIT.

### XKR5 is highly expressed in hematopoietic cells

**Fig. 3:**
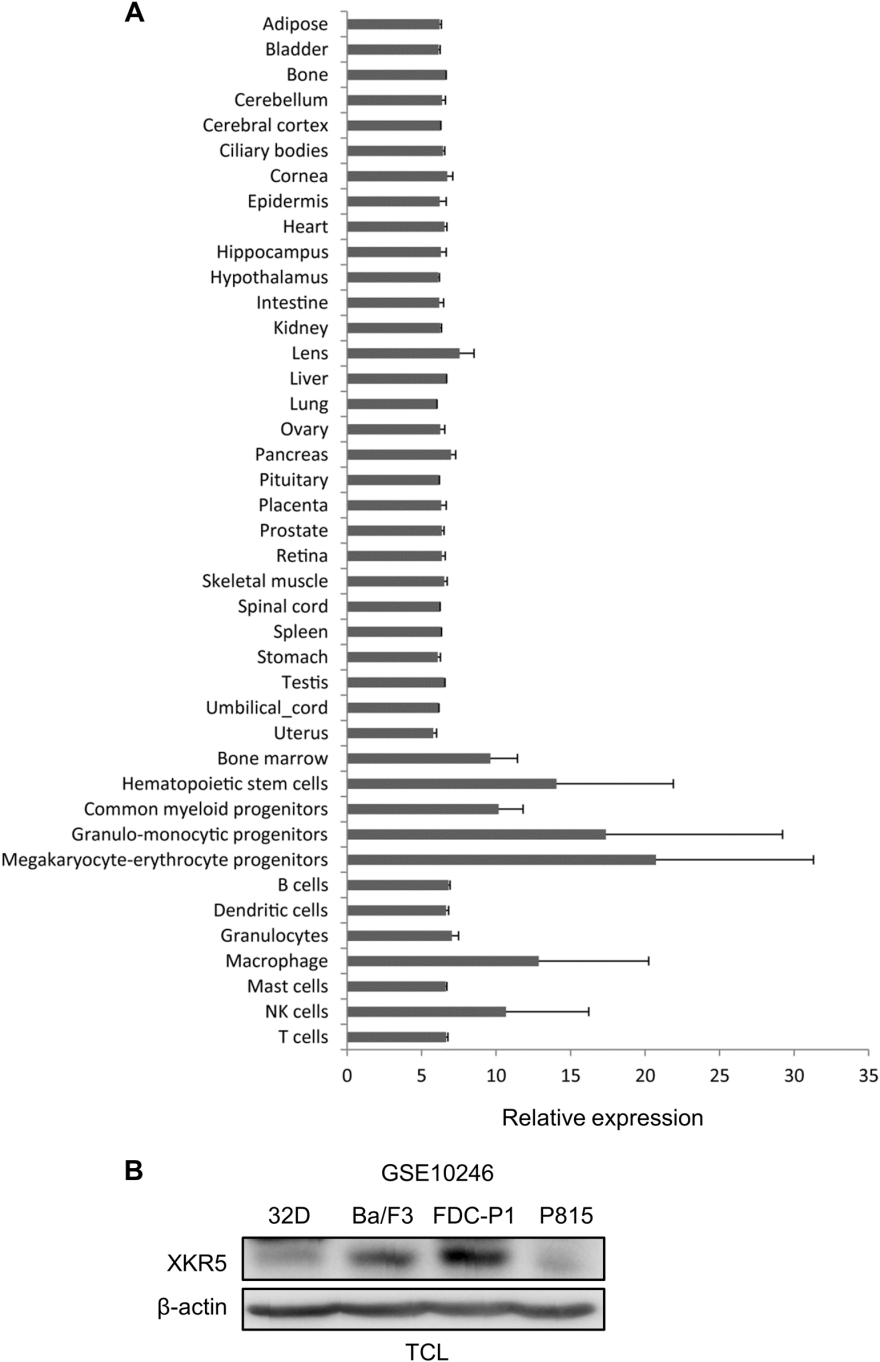
Tissue distribution of XKR5 expression. **a** Gene expression dataset GSE10246 was downloaded from PubMed and the expression of XKR5 in various tissues was extracted and analyzed by Graphpad. **b** 32D, Ba/F3, FDC-P1, and P815 cells were lysed. The total cell lysates were separated by SDS-PAGE, electrotransferred and probed with an antibody against XKR5

XKR5 is a novel, hitherto uncharacterized protein and its biological role is unknown. In order to analyze the expression of XKR5 in different tissues and whether it is expressed in tissues in which KIT/D816V often is expressed, we analyzed the gene expression dataset GSE10246. Interestingly, we observed high XKR5 expression in hematopoietic cells compared to other tissues (Fig. 3a). In addition, Western blot analysis of hematopoietic cells showed strong expression of XKR5 (Fig. 3b). Since the KIT/D816V mutation has been reported in several hematological malignancies, including mastocytosis and core binding factor acute myeloid leukemia (CBF AML), this suggests that the interaction found is relevant for KIT function.

### XKR5 is phosphorylated by KIT/D816V on Tyr 369, Tyr487, and Tyr 543

**Fig. 4:**
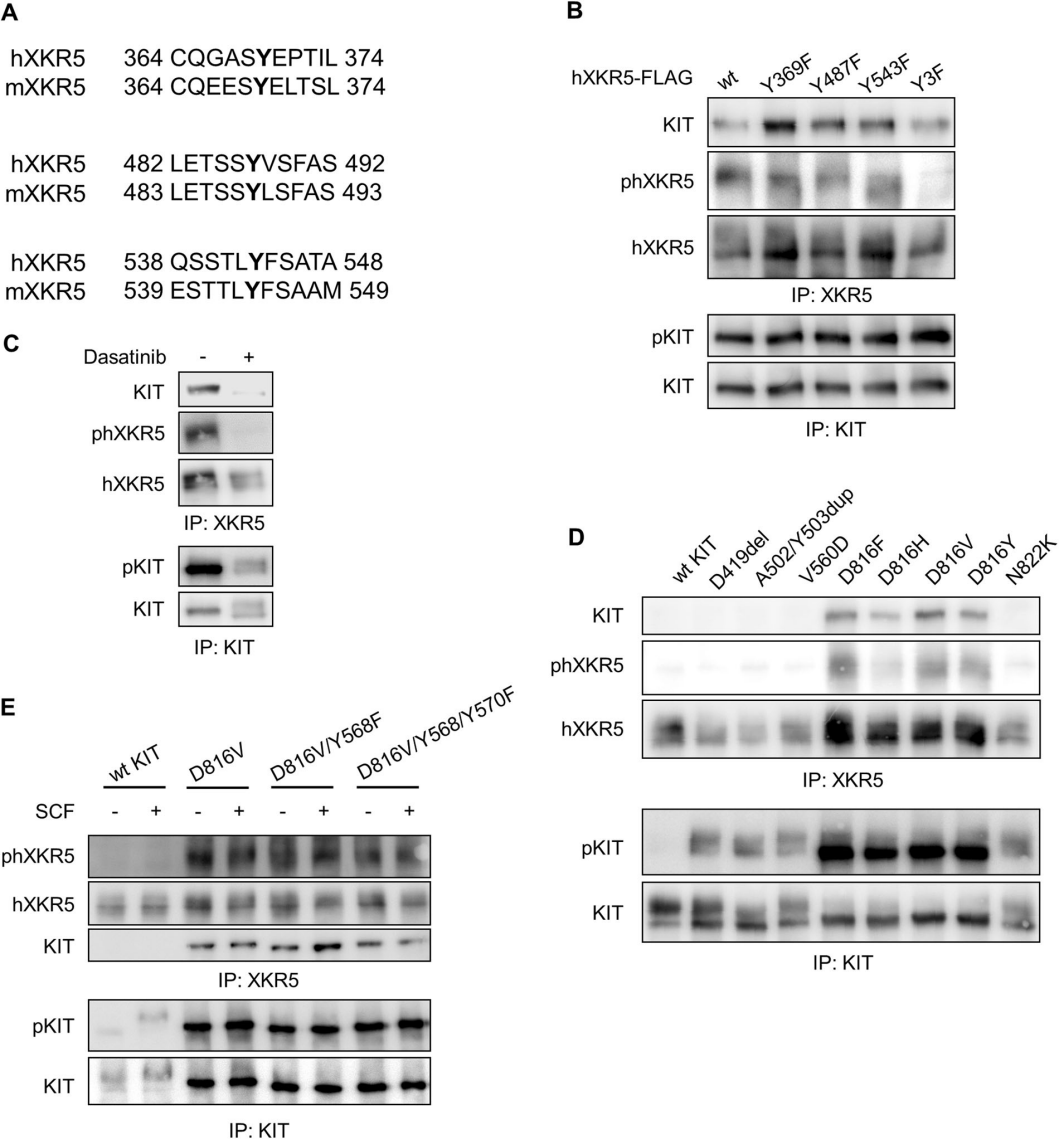
Tyrosines 369, 487, and 543 are conserved between murine and human XKR5 and essential for the function of XKR5. **a** Alignment of the protein sequence of human and murine XKR5 show that Tyr369, Tyr 487, and Tyr 543 are conserved between murine and human XKR5. **b** COS1 cells were transfected with KIT/D816V and wild-type XKR5 or triple Y-to-F mutant of XKR5. Cells were lysed and immunoprecipitated with KIT or FLAG antibodies (to detect XKR5), followed by SDS-PAGE and western blotting analysis. **c** COS1 cells were co-transfected with KIT/D816V and wild-type XKR5, and incubated with the kinase inhibitor dasatinib (2 nM) for 5 h. Cells were lysed and immunoprecipitated with KIT or FLAG antibodies, to detect XKR5, followed by SDS-PAGE and western blotting analysis. **d** COS1 cells were co-transfected with XKR5 and either wild-type KIT or KIT mutants. Cells were lysed and immunoprecipitated with KIT or FLAG antibodies (to detect XKR5), followed by SDS-PAGE and western blotting analysis. **e** COS1 cells were co-transfected with XKR5 and either wild-type KIT, KIT/D816V, or KIT/D816V/Y568F/Y570F (lacking SRC binding sites). Cells were lysed and immunoprecipitated with KIT or FLAG antibodies (to detect XKR5), followed by SDS-PAGE and western blotting analysis

KIT/D816V differs from wild-type KIT in several aspects. While both are active tyrosine kinases, they display different substrate specificity^5^. We have recently reported that KIT/D816V, but not ligand stimulated wild-type KIT, induces tyrosine phosphorylation of p110delta and the adaptor protein SLAP, and that the phosphorylation of these proteins contribute to KIT/D816V-mediated cell transformation^2,4^. To know whether KIT/D816V also holds similar properties to XKR5, we first checked tyrosine phosphorylation of XKR5 using a pan-phosphotyrosine antibody, 4G10. The results show that both murine and human XKR5 are tyrosine phosphorylated by KIT/D816V but not by wild-type KIT (Fig. 2a). Since both murine and human XKR5 were tyrosine phosphorylated, we speculated that the tyrosine phosphorylation sites in the two species are likely to be conserved. Sequence alignment of the XKR5 sequences demonstrated that three tyrosine residues in the intracellular domain of XKR5 are conserved between murine and human XKR5 including Tyr369, Tyr 487, and Tyr 543 (Fig. 4a). We generated a panel of XKR5 Y-to-F mutants of these sites and observed that each single mutation of those three sites reduced tyrosine phosphorylation of XKR5 and that the triple mutation almost completely eliminated tyrosine phosphorylation (Fig. 4b). Therefore, we suggest that Tyr369, Tyr 487, and Tyr 543 in XKR5 are phosphorylated by KIT/D816V. Furthermore, we showed that phosphorylation of XKR5 can be blocked by the KIT/D816V inhibitor dasatinib (Fig. 4c).

### XKR5 is phosphorylated by D816X mutants of KIT but not by other oncogenic KIT mutants

KIT mutations commonly occur in GISTs, mastocytosis, CBF AML, and less commonly in melanoma, germ cell tumors and other malignancies^1,6^. The D816V mutation in exon 17 of KIT, which encodes the activation loop of the kinase domain, is the most commonly occurring KIT mutation in mastocytosis and CBF AML. In GISTs, mutations in KIT are usually localized to exon 11, which encodes the juxtamembrane region, and less often to exon 9, 13 and 17. To understand whether phosphorylation and association of XKR5 is common to all activating KIT mutants, we co-expressed XKR5 with KIT mutated at exon 9, 11, and 17, as well as other D816X mutants, apart from D816V. Immunoprecipitation of XKR5 demonstrated that all KIT D816X mutants can bind to and phosphorylate XKR5, whereas the other KIT mutants neither associated with nor phosphorylated XKR5 (Fig. 4d). Thus, association of KIT with XKR5 is limited to the D816X mutants.

### The tyrosine phosphorylation of XKR5 is not dependent on SRC family kinases

SRC family kinases play a crucial role in the activation of wild-type KIT^7^ while KIT/D816V gains SRC-like kinase activity and becomes independent of SRC family kinases for the activation of KIT/D816V and its downstream signaling^5^. Tyr 568 in KIT is the binding site of SRC family kinases and the binding can be further enhanced by phosphorylation of Tyr 570^8^. Co-expression of XKR5 with KIT/D816V carrying mutations of the SRC family kinases binding sites did not block tyrosine phosphorylation of XKR5 nor the association between XKR5 and KIT/D816V (Fig. 4e), indicating that SRC family kinases are not necessary for phosphorylation of XKR5 by KIT/D816V.

### Tyrosine phosphorylation of XKR5 leads to inhibition of signal transduction pathways downstream of KIT/D816V

**Fig. 5:**
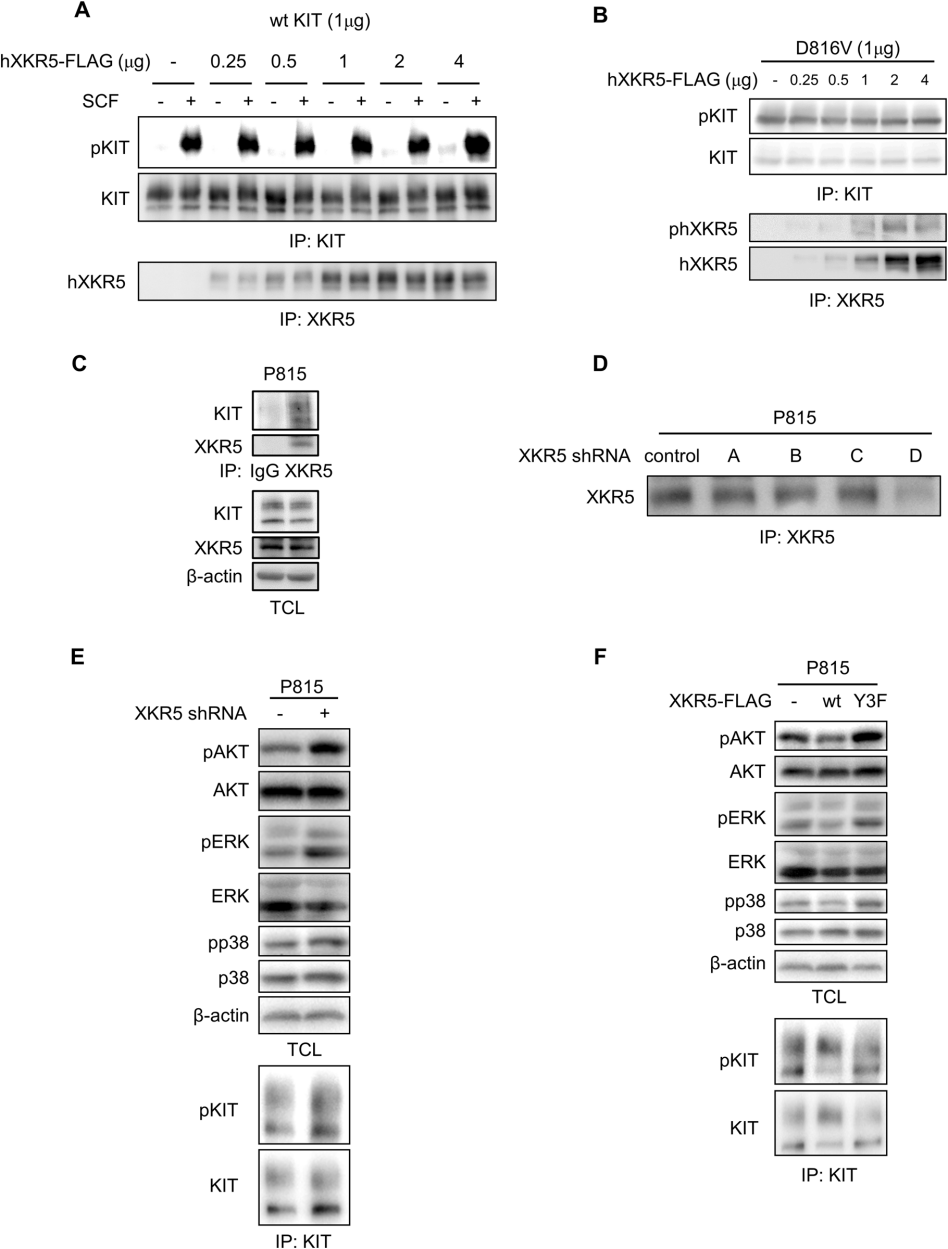
XKR5 does not influence the kinase activity of KIT but is a negative regulator of the downstream signaling molecules ERK, AKT, and p38. **a** COS1 cells were co-transfected with wild-type KIT and various amounts of XKR5. Cells were lysed and immunoprecipitated with KIT or FLAG antibodies, followed by SDS-PAGE and western blotting analysis. **b** COS1 cells were co-transfected with KIT/D816V and various amounts of XKR5. Cells were lysed and immunoprecipitated with KIT or FLAG antibodies, followed by SDS-PAGE and western blotting analysis. **c** P815 cells were lysed and immunoprecipitated with XKR5 antibody or rabbit IgG, followed by SDS-PAGE and western blotting analysis. **d** P815 cells were stably transfected with four different shRNAs targeting XKR5. Cells were lysed and immunoprecipitated with XKR5 antibodies, followed by SDS-PAGE and western blotting analysis to detect the level of XKR5 protein. **e** P815 cells were stably transfected with shRNA targeting XKR5 or control shRNA. Cells were lysed, followed by SDS-PAGE and western blotting analysis using antibodies against phosphoAKT, phosphoERK and phosphop38 and the corresponding non-phosphorylated proteins, respectively. **f** P815 cells were transfected with wild-type XKR5 or the Y-to-F triple mutant of XKR5. Cells were lysed and total cell lysates were probed with antibodies against phosphoAKT, phosphoERK and phosphop38 and the corresponding non-phosphorylated proteins, respectively. In parallel, lysates were imunoprecipitated with KIT antibody followed by SDS-PAGE and western blotting analysis

In order to investigate the role of XKR5 in KIT/D816V signaling, we examined whether XKR5 regulates KIT activation. Co-expression of KIT and XKR5 showed that the activation of both wild-type KIT and KIT/D816V remains unaffected by the presence or absence of XKR5 (Fig. 5a,b), suggesting that it, despite associating with KIT, does not influence its kinase activity.

To further investigate whether XKR5 regulates signal transduction pathways downstream of KIT/D816V, the murine mastocytoma cell line P815, which endogenously expresses KIT/D814V (corresponding to KIT/D816V in humans), was used as cell model. Immunoprecipitation with XKR5 antibody showed that KIT/D814V binds to XKR5 in P815 cells (Fig. 5c). The expression of XKR5 was knocked down by three different shRNAs and the one that gave best knockdown was used for further experiments (Fig. 5d). Knockdown of XKR5 in P815 cells enhanced phosphorylation of the three downstream signaling molecules AKT, ERK, and p38 that are important for cell survival and proliferation (Fig. 5e). In line with that, overexpression of XKR5 inhibited phosphorylation of AKT, ERK and p38 (Fig. 5f), indicating that XKR5 is a negative regulator of KIT/D816V signaling. To investigate whether the inhibition of KIT/D816V signaling by XKR5 is mediated by the tyrosine phosphorylation of XKR5, the triple mutant of XKR5 lacking the three phosphorylation sites was overexpressed in P815 cells. As shown in Fig. 5f, expression of the tyrosine mutant of XKR5 enhanced phosphorylation of AKT, ERK, and p38, suggesting that the inhibition of KIT/D816V signaling by XKR5 is mediated through tyrosine phosphorylation of XKR5.

### Tyrosine phosphorylation of XKR5 leads to inhibition of KIT/D816V-mediated cell proliferation and colony formation in semi-solid medium

**Fig. 6:**
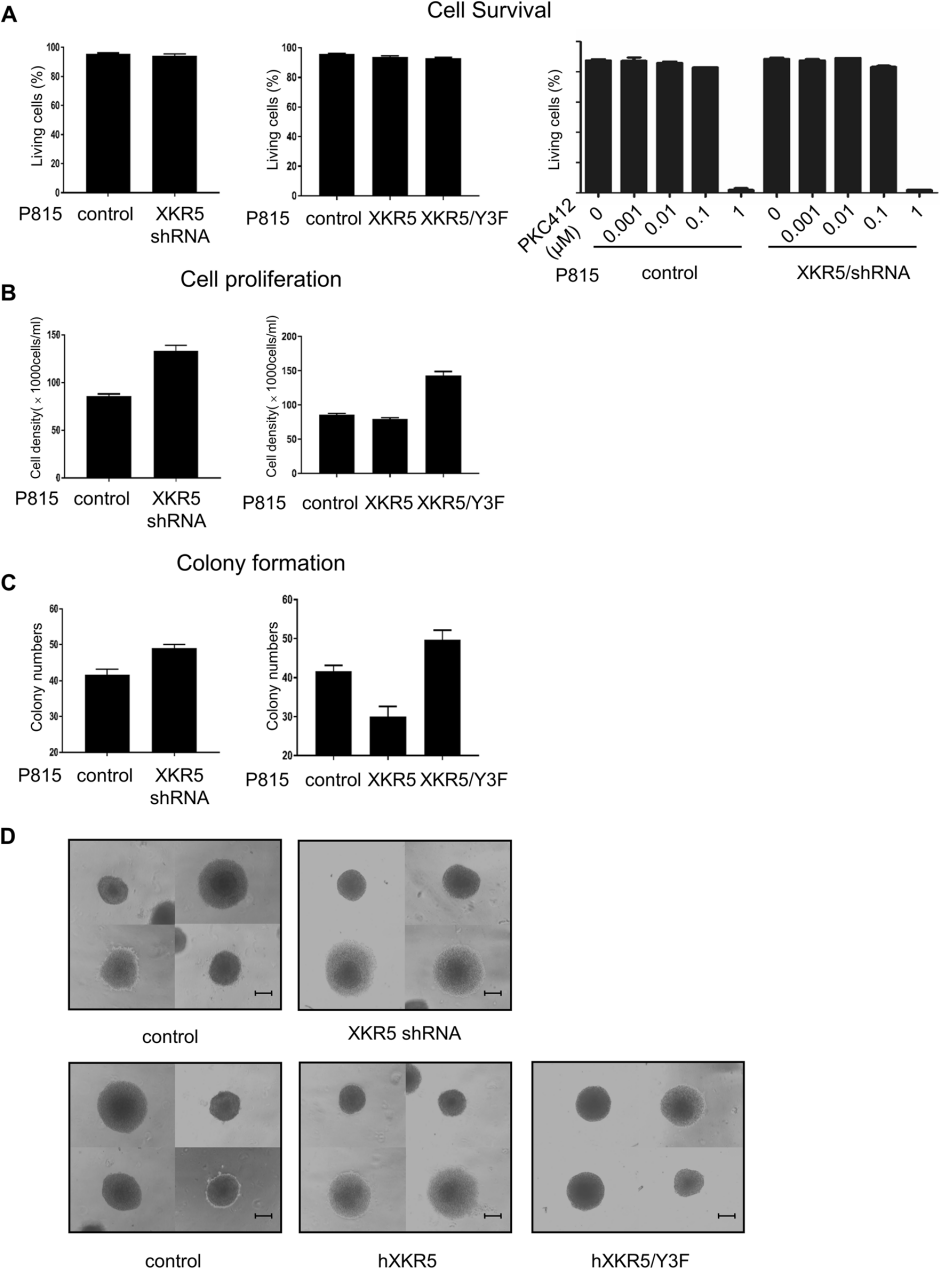
XKR5 does not influence survival of P815 cells, but is a negative regulator of both proliferation and the capacity to form colonies in semi-solid medium. P815 cells were seeded in 24-well plates at a density of 70,000 cells/ml and incubated for two days. **a** Cells were stained with cell apoptosis detection kit and analyzed by flow cytometry to detect the level of cell survival. **b** The number of live cells was counted by trypan blue exclusion to measure the cell proliferation. **c** P815 cells were mixed with semi-solid colony assay medium MethoCult® M3231 and 100 cells were seeded in 24-well plates. After incubation for 6 days, colonies were counted. Statistical significance was calculated by *t* test. **d** Photographs of colonies after 6 days of incubation, the scale bar indicates 100 μm

Due to the inhibitory role of XKR5 on the activation of AKT, ERK, and p38, we further examined whether XKR5 also is a negative regulator of KIT/D816V-mediated cell transformation. By flow cytometry, we found that neither knockdown nor overexpression of XKR5 changed the survival of P815 cells, even in the presence of KIT inhibitor PKC412 (Fig. 6a), while knockdown of XKR5 enhanced the proliferation and overexpression of XKR5 inhibited proliferation of P815 cells. Furthermore, overexpression of the triple Y-to-F mutant of XKR5 enhanced proliferation of P815 cells (Fig. 6b). Taken together, these data suggest that XKR5 is a negative regulator of KIT/D816V-mediated proliferation and that it is mediated through tyrosine phosphorylation of XKR5. In a similar fashion, knockdown of XKR5 enhanced colony formation of P815 cells in semi-solid medium, while overexpression of XKR5 suppressed colony formation. Similar to the effect on cell proliferation, the effect was dependent on tyrosine phosphorylation of XKR5 (Fig. 6c, d). Taken together, these results suggest that XKR5 is a novel phosphorylation-dependent negative regulator of KIT/D816V-mediated cell transformation.

## Discussion

Mutated constitutively active receptor tyrosine kinases are commonly found in human malignancies. One of the most common mutants of KIT in human tumors is the KIT/D816V mutant. It is characterized by a high constitutive kinase activity, but also by an altered substrate specificity^3,5^. Together with the fact that KIT/D816V is trapped in the Golgi and signals from there^9^, it makes it likely that several signaling proteins are activated by KIT/D816V but not by wild-type KIT. We have so far identified p110 delta and SLAP as proteins phosphorylated only by KIT/D816V and not by wild-type KIT^2,4^. In order to identify additional KIT/D816V-specific substrates, we immunoprecipitated KIT from KIT/D816V-expressing Ba/F3 cells, ran out the immunoprecipitates on SDS-PAGE and stained with Coomassie Brilliant Blue. Bands of interest were excised and analyzed by mass spectrometry. Several proteins were identified, of which the band identified as XKR5 was the most prominent.

XKR5 is a by and large uncharacterized protein belonging to the XKR group of proteins that are related to the Kell blood group antigen. The family of proteins consists of 9 different family members, XKR1-9. They are predicted to be transmembrane proteins with six transmembrane regions^10^. XKR1 has been shown to regulate cell volume by transport of divalent cations^11^. Other members, such as XKR4, XKR8, and XKR9, are involved in facilitation of apoptotic phosphatidylserine exposure^10^. One recent genome wide association study correlated the expression of XKR9 with erythrocyte cadmium^12^, suggesting a possible role in cadmium absorption and/or metabolism. It is likely that all members are involved in some type of transportation across the plasma membrane.

Interaction of XKR5 with KIT leads to phosphorylation of XKR5 on Tyr 369, Tyr487, and Tyr 543. Tyrosine phosphorylated XKR5 acts as a negative regulator of KIT signaling, which leads to downregulation of phosphorylation of ERK, AKT, and p38. The biological effects are reduced proliferation and reduced colony forming capacity in semi-solid medium. The mechanism by which XKR5 exerts its effect is not known. It is likely that the three phosphorylation sites constitute docking sites for proteins involved in signaling downstream of XKR5. However, the sequence surrounding the tyrosine residues do not give any obvious hint as to which proteins might be involved. Future studies will look into the details of how XKR5 exerts its negative effect on signaling.

Transformation by oncogenic mutants of receptor tyrosine kinases involves the activation of a number of signal transduction molecules, both those that mediate a positive signal but also an activation of proteins that are negative regulators. In leukemic cells transformed by the related RTK FLT3, we have previously demonstrated that activation of FLT3 strongly induces SOCS6 expression^13^. It might seem counter intuitive to think that the upregulation of a negative regulator could be important for transformation, but there are several cases where too high activity of a receptor tyrosine kinase has been shown to induce apoptosis, rather than transformation. E.g., A431 cells that overexpress the EGF receptor respond to EGF stimulation by induction of apoptosis^14^. Furthermore, ectopic expression of FLT3 has been shown to induce apoptosis in certain cell types^15^. Thus, engagement of a negative regulator such as XKR5 could be important for transformation.

Taken together, our data demonstrate that XKR5 is a novel type of negative regulator of KIT mediated transformation that influences AKT, ERK, and p38 signaling, as well as cell proliferation and transformation but not apoptosis.

## Materials and Methods

### Cytokines and antibodies

Recombinant human stem cell factor (SCF) was from Prospec Tany, Israel. The rabbit antibody KitC1 that recognizes the C-terminal tail of human KIT was purified as described^16^. The antibody against murine XKR5 was generated by immunizing rabbit with a synthetic peptide corresponding to the C-terminus of XKR5 (CHHAAVGVWVSLPQ) conjugated to KLH and affinity purified. Antibodies against AKT (SC8312), ERK (SC292838) and pERK (SC16982-R) were from Santa Cruz Biotechnology. pAKT antibody (cat. no. 2118-1) was from Epitomics. Phosphotyrosine antibody 4G10 (cat. no. 05-321) was from Millipore. pp38 (cat. no. 612289) and p38 antibodies (cat. no. 612169) were from BD Biosciences. β-actin (cat. no. A1978) and FLAG antibodies (cat. no. F1804) were from Sigma-Aldrich. PE labeled KIT antibody (104D2; cat. no. 313204 was from BioLegend. Horseradish peroxidase conjugated anti-rabbit (cat. no. 31460) and anti-mouse (cat. no. 31430) secondary antibodies were from Life Technologies.

### Kits and reagents

ShRNAs were from Origene. QuikChange mutagenesis kit was from Agilent Technologies. Annexin V-PE apoptosis detection kit was from BD Biosciences. Transfection reagent Lipofectamine 2000, Coomassie Blue and protein G Dyna beads for immunoprecipitation were from Life Technologies. Chemiluminescent HRP substrate was from Millipore. Aprotinin and phenylmethylsulfonyl fluoride were from Sigma-Aldrich. All the reagents and kits were used according to the manufacturer’s instructions.

### Cell culture

Ba/F3 cells (DSMZ, Braunschweig, Germany) were grown in RPMI 1640 medium supplemented with 10% heat-inactivated fetal bovine serum, 100 units/ml penicillin and 100 µg/ml streptomycin, and 10 ng/ml recombinant murine IL-3 as previously described^17^. The virus packaging cell line EcoPack and the mastocytoma cell line P815 were grown in Dulbecco’s modified Eagle’s medium supplemented with 10% fetal bovine serum, 100 units/ml penicillin and 100 µg/ml streptomycin. FDC-P1 cells were grown in Dulbecco’s modified Eagle’s medium supplemented with 10% fetal bovine serum, 100 units/ml penicillin and 100 µg/ml streptomycin, and 10 ng/ml recombinant murine IL-3. In order to establish Ba/F3 cell lines expressing KIT, EcoPack cells were transfected with KIT constructs in pMSCVpuro vector. Supernatants were collected to infect Ba/F3 cells followed by 2-weeks selection in 1.2 μg/ml puromycin. Expression of KIT was confirmed by flow cytometry and immunoblotting. KIT expressing Ba/F3 cells were grown in the same medium as untransfected Ba/F3 cells. All cells were regularly tested for mycoplasma.

### Large scale purification of KIT and associated proteins

900 million Ba/F3 cells expressing wild-type KIT or KIT/D816V were starved in serum-free medium in the absence of IL-3 for 4 h followed by SCF stimulation (100 ng/ml) for 2 min. Cells were washed once in ice-cold PBS and lysed in 30 ml lysis buffer containing 1% Triton X-100, 25 mM Tris, pH 7.5, 150 mM NaCl, 5 mM EDTA, 10% glycerol, 2 µg/ml aprotinin, 1 mM Na_3_VO_4_ and 1 mM phenylmethylsulfonyl fluoride. The lysates were centrifuged at 14,000 × *g* for 15 min at 4 °C and supernatants were incubated end-over-end with 10 μg KitC1 antibody for 1 h followed by incubation with 100 μl protein G Dyna beads for 30 min at 4 °C. The immunoprecipitates were washed three times in lysis buffer, boiled for 5 min in SDS-PAGE sample buffer and separated by SDS-PAGE followed by staining with Coomassie Blue. Band of interest were excised and identified by mass spectrometry.

### Mass spectrometry

The gel bands of interest were excised from the gel, and subjected to in-gel digestion by trypsin (sequencing grade; Promega) as described previously^18^. The resulting peptides were purified using TiO_2_ chromatography^19,20^ and desalted^20^ prior to mass spectrometry (MS) analysis. The purified peptides were applied onto an EASY nano-LC system (Proxeon Biosystems, Odense, Denmark), and analyzed using a LTQ Orbitrap XL mass spectrometer (Thermo Electron, Bremen, Germany). The peptides were concentrated on a 1.0-cm precolumn (75-μm inner diameter, 360-μm outer diameter, ReproSil-Pur C_18_ AQ 3 μm (Dr. Maisch, Ammerbuch-Entringen, Germany)) and eluted from the precolumn using a gradient from 100% phase A (0.5% acetic acid aqueous solution) to 40% phase B (0.5% acetic acid, 80% acetonitrile) at 200 nl min^−1^ directly onto an 8-cm analytical column (50-μm inner diameter, 360-μm outer diameter, ReproSil-Pur C_18_ AQ 3 μm). The instrument was operated in a data-dependent mode automatically switching between MS, MS^2^, and pdMS^3^ ^21^. The data were processed using Proteome Discoverer, beta-version 1.1.0.228. The files were subsequently submitted to an in-house MASCOT server (version 2.2.05) (Matrix Science Ltd., London, U.K.) for database searching through the Proteome Discoverer program. The data were searched against the Swiss-Prot mouse sequence database. The search was performed choosing trypsin as specific enzyme. A maximum of 2 missed cleavages were allowed. Carbamidomethyl (C) was chosen as fixed modification. As variable modifications Oxidation (M) were chosen. The data were searched with a peptide mass tolerance of 10 p.p.m. and a fragment mass tolerance of 1.1Da. Finally, the data were searched against the decoy database to calculate a false discovery rate (FDR) of 1 and 5% using the Proteome Discoverer program. Protein grouping was performed in the Proteome Discoverer program.

### Analysis of XKR5 expression in tissues

GEO dataset GSE10246 was downloaded from PubMed and the expression of XKR5 in various tissues was extracted and analyzed by the software Graphpad.

### Cell stimulation, immunoprecipitation, and western blotting

Cell stimulation, immunoprecipitation, and western blotting was performed as previously described^4^.

### Co-localization of XKR5 and KIT

COS1 cells were co-transfected with FLAG-tagged XKR5 and wild-type KIT or KIT/D816V. 48 h after transfection, cells were stimulated with SCF for 2 min. Cells were then fixed in 4% para-formaldehyde in PBS for 30 min. Blocking and permeabilization was done by adding a mixture of 0.5% Triton-X100 in PBS and 5% goat serum. Cells were then stained with antibody and washed before examination by confocal microscopy. Co-localization was measured with CoLocalizer Pro 2.7.1 software.

### Cell survival and proliferation assay

Cell survival and proliferation assay was performed as described^4^.

### Colony formation assay

P815 cells were mixed with semi-solid colony assay medium MethoCult® M3231 (Stem cell technologies) according to the manufacturer’s instruction. Cell mixture was seeded in 24-well plates. After incubation for 6 days, colonies were counted and photographed. Statistical significance was calculated by *t* test.

## References

[1] Lennartsson J, Rönnstrand L (2012). Stem cell factor receptor/c-Kit: from basic science to clinical implications. Physiol. Rev..

[2] Kazi JU, Agarwal S, Sun J, Bracco E, Rönnstrand L (2014). Src-like-adaptor protein (SLAP) differentially regulates normal and oncogenic c-Kit signaling. J. Cell Sci..

[3] Piao X, Paulson R, van der Geer P, Pawson T, Bernstein A (1996). Oncogenic mutation in the Kit receptor tyrosine kinase alters substrate specificity and induces degradation of the protein tyrosine phosphatase SHP-1. Proc. Natl Acad. Sci. USA.

[4] Sun J (2014). The PI3-kinase isoform p110delta is essential for cell transformation induced by the D816V mutant of c-Kit in a lipid-kinase-independent manner. Oncogene.

[5] Sun J, Pedersen M, Rönnstrand L (2009). The D816V mutation of c-Kit circumvents a requirement for Src family kinases in c-Kit signal transduction. J. Biol. Chem..

[6] Lennartsson J, Rönnstrand L (2006). The stem cell factor receptor/c-Kit as a drug target in cancer. Curr. Cancer Drug Targets.

[7] Voytyuk O (2003). Src family kinases are involved in the differential signaling from two splice forms of c-Kit. J. Biol. Chem..

[8] Lennartsson J (1999). Phosphorylation of Shc by Src family kinases is necessary for stem cell factor receptor/c-kit mediated activation of the Ras/MAP kinase pathway and c-fos induction. Oncogene.

[9] Xiang Z, Kreisel F, Cain J, Colson A, Tomasson MH (2007). Neoplasia driven by mutant c-KIT is mediated by intracellular, not plasma membrane, receptor signaling. Mol. Cell Biol..

[10] Suzuki J, Imanishi E, Nagata S (2014). Exposure of phosphatidyserine by Xk-related protein familymembrers during apoptosis. J. Biol. Chem..

[11] Rivera A, Kam SY, Ho M, Romero JR, Lee S (2013). Ablation of the Kell/Xk complex alters erythrocyte divalentcation homeostasis. Blood Cells Mol. Dis..

[12] Borné Y (2016). Genome wide association study identifies two loci associated with cadmium in erythrocytesamong never-smokers. Hum. Mol.Genet.

[13] Kazi JU (2012). Suppressor of cytokine signaling 6 (SOCS6) negatively regulates Flt3 signal transduction through direct binding to phosphorylated tyrosines 591 and 919 of Flt3. J. Biol. Chem..

[14] Gulli LF, Palmer KC, Chen YQ, Reddy KB (1996). Epidermal growth factor-induced apoptosis in A431 cells can be reversed by reducing the tyrosine kinase activity. Cell Growth Differ..

[15] Oveland E (2012). Ectopic expression of Flt3 kinase inhibits proliferation and promotes cell death in different human cancer cell lines. Cell Biol. Toxicol..

[16] Blume-Jensen P, Siegbahn A, Stabel S, Heldin CH, Rönnstrand L (1993). Increased Kit/SCF receptor induced mitogenicity but abolished cell motility after inhibition of protein kinase C. EMBO J..

[17] Kazi JU, Sun J, Rönnstrand L (2013). The presence or absence of IL-3 during long-term culture of Flt3-ITD and c-Kit-D816V expressing Ba/F3 cells influences signaling outcome. Exp. Hematol..

[18] Nawrocki A (1998). Correlation of acidic and basic carrier ampholyte and immobilized pH gradient two-dimensional gel electrophoresis patterns based on mass spectrometric protein identification. Electrophoresis.

[19] Thingholm T. E., Bak S., Beck-Nielsen H., Jensen O. N., Gaster M. Characterization of human myotubes from type 2 diabetic and nondiabetic subjects using complementary quantitative mass spectrometric methods. *Mol. Cell Proteomics*. **10****:** M110 006650 (2011). 10.1074/mcp.M110.006650 PMC3186194 21697546

[20] Thingholm TE, Larsen MR (2016). The use of titanium dioxide for selective enrichment of phosphorylated peptides. Methods Mol. Biol..

[21] Beausoleil SA (2004). Large-scale characterization of HeLa cell nuclear phosphoproteins. Proc. Natl Acad. Sci. USA.

